# Smart breeding approaches in post-genomics era for developing climate-resilient food crops

**DOI:** 10.3389/fpls.2022.972164

**Published:** 2022-09-16

**Authors:** Rubab Zahra Naqvi, Hamid Anees Siddiqui, Muhammad Arslan Mahmood, Syed Najeebullah, Aiman Ehsan, Maryam Azhar, Muhammad Farooq, Imran Amin, Shaheen Asad, Zahid Mukhtar, Shahid Mansoor, Muhammad Asif

**Affiliations:** Agricultural Biotechnology Division, National Institute for Biotechnology and Genetic Engineering, Pakistan Institute of Engineering and Applied Sciences, Faisalabad, Pakistan

**Keywords:** climate change, agriculture, genomics, breeding, food crops

## Abstract

Improving the crop traits is highly required for the development of superior crop varieties to deal with climate change and the associated abiotic and biotic stress challenges. Climate change-driven global warming can trigger higher insect pest pressures and plant diseases thus affecting crop production sternly. The traits controlling genes for stress or disease tolerance are economically imperative in crop plants. In this scenario, the extensive exploration of available wild, resistant or susceptible germplasms and unraveling the genetic diversity remains vital for breeding programs. The dawn of next-generation sequencing technologies and omics approaches has accelerated plant breeding by providing the genome sequences and transcriptomes of several plants. The availability of decoded plant genomes offers an opportunity at a glance to identify candidate genes, quantitative trait loci (QTLs), molecular markers, and genome-wide association studies that can potentially aid in high throughput marker-assisted breeding. In recent years genomics is coupled with marker-assisted breeding to unravel the mechanisms to harness better better crop yield and quality. In this review, we discuss the aspects of marker-assisted breeding and recent perspectives of breeding approaches in the era of genomics, bioinformatics, high-tech phonemics, genome editing, and new plant breeding technologies for crop improvement. In nutshell, the smart breeding toolkit in the post-genomics era can steadily help in developing climate-smart future food crops.

## Climate change scenario and its effect on agriculture and food security

The world’s population is gradually up-surging and the biggest challenge is food security. The other challenges include climate change and population growth (Abberton et al., 2016). The increasing population demands more food and exerts an extra burden on agricultural resources (Ray et al., 2013). Climate change is one of the biggest challenges in the sustainable production of agricultural crops. It is defined as “the significant changes in the different elements of metrology such as temperature and precipitation, for which averages have been computed over a long period” (Malhi et al., 2021). For the past few decades, the major cause of devastating climate change is the human activities that altered the global atmospheric composition. In the troposphere where life exists, the atmospheric greenhouse effect occurs. Other causes involve rapid industrialization, urbanization, scorching of farming wastes, deforestation, and use of non-degradable merchandises, which pose a serious threat to the sustainable environment. Climate change has evoked variations in temperature, rainfall, and atmospheric conditions that adversely affect the developmental, morphological, cellular, and molecular mechanisms in plants. It can affect the crop production by direct, indirect, and socio-economic means. For example, direct effects such as morphological, physiological, and phenotypic changes in the plant productivity. Indirect effects include soil fertility, rise in the sea level, pest pressure, and availability of irrigation whereas the socio-economic effects consist of food demand, costs, trading, and unequal distribution. These factors can severely influence the agricultural production.

Since 1750, the concentration of greenhouse gases such as nitrous oxide (N_2_O), carbon dioxide (CO_2_), and methane (CH_4_) have been significantly increased by 20, 40, and 150%, respectively. The mainly contributing greenhouse gas is CO_2_ which has a positive effect on the plant growth through CO_2_ fertilization (Wang S. et al., 2020). Enhanced CO_2_ directly influences the photosynthesis, exchange of gases, and numerous other developmental processes in plants (Gray and Brady, 2016). Simultaneously, the nutritional value, as well as the quality of food decreases in response to sharp CO_2_ in the atmosphere that is caused by various other environmental factors. However, long-term exposure of plants to elevated CO_2_ can decrease the photosynthesis because of photosynthetic acclimation, ultimately affecting the vegetable quality in plants (Dong et al., 2018). Recently, Parvin et al. (2019) identified the reduced concentration of Fe, Zn, S, and P in lentil and faba bean crops upon high CO_2_ conditions. It has been observed that over the past 30 years, there is a decline in CO_2_ fertilization due to the lower availability of water and shifting nutrient concentrations (Wang S. et al., 2020).

**FIGURE 1:**
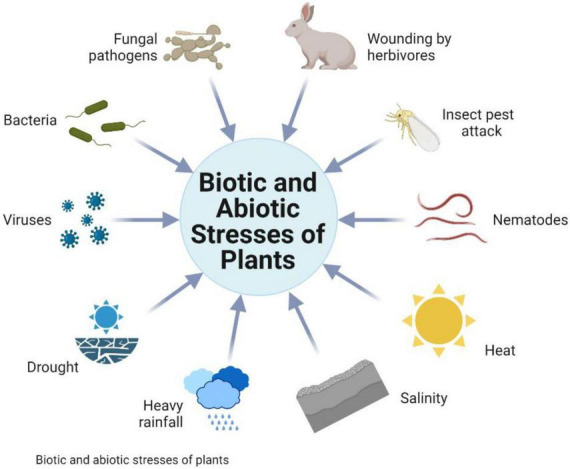
Different types of various stresses in crop plants.

Several biotic and abiotic stresses hit the crops’ productivity (Figure 1), which are becoming severe due to climate change. Due to extreme temperature, wheat production is heavily affected in various countries and may reduce the crop yield by 6% for every °C rise in temperature. In cereal crops like wheat, drought and high temperature are the key factors with a high impact on yield and *Rubisco*, the main photosynthetic enzyme. If the temperature increases from 35°C, it stops the photosynthetic process (Barnabás et al., 2008).

The combined impact of drought and high temperature has been observed to result in more damaging outcomes as compared to the individual stress (Wang and Huang, 2004). The global yield of important crops like wheat and maize has been decreased from 1981 to 2010 relative to the prior years (Iizumi et al., 2018). It is estimated that atmospheric CO_2_ will increase up to ∼730–1000 ppm by the end of 21st century which is allied with the peaked mean global temperature that will ultimately bring the significant changes in global climate (Balasooriya et al., 2018). Elevation of temperature accelerates the metabolic activities of insect pests and enhances their crop damage frequency. Besides it, the preeminent levels of CO_2_ make the food crops vulnerable to insects and different pathogens. Overall, the effect of climate change on crops remains detrimental. The dilemma becomes worse because of the fluctuations in annual rainfall, temperature, and various environmental factors which are directly associated with climate change.

## Conventional and modern molecular breeding for crop improvement

Humans have used an artificial selection of plants for the past 10,000 years to select the crop plants for desired traits *via* breeding. Conventional agricultural procedures are intended to improve the plants yield and their nutritional composition but recent modern methods offer a vast choice of options and innovations in the breeding methods. These newly developed methods can be used to cope with the devastating plant biotic as well as abiotic stresses and to combat the growing demand for food commodities (Supplementary Table 1).

Crop breeding revolutionized when Mendelian laws were announced. With the invention of new cutting-edge genomics tools, crop development is modified greatly. In this decade, novel techniques, e.g., genomic selection, modern speed breeding, and high-throughput crop phenotyping (HTTP) have been shown to speed up the plant breeding mechanism. Biotechnological interventions for instance genetic engineering tools (gene transformation) have also played an important role in the development of crops having desirable traits. Besides this, other techniques such as whole genome sequencing (WGS), genomics, gene identification, gene isolation, and fast molecular markers are opted to be a good strategy for improving cisgenesis, intragenesis, mutation, and polyploidy breeding (Muth et al., 2008; Murovec et al., 2017).

Crop breeding is a decision-making process at all its stages of breeding program such as testing, mapping, and introgression of traits, where breeders select the individual plants from large segregating populations harboring the best traits (Kaiser et al., 2020). But these conventional breeding practices mostly remain very slow and inadequate to enhance the development of crop varieties. Conventionally once the crosses are made among two parent plants, the successive generations are achieved for the identification of superior individual. The whole process involves the plants multi-years testing in replicated field trials at multiple locations for the detection of genetic potentional of candidate genotype across a wide range of conditions (Voss-Fels et al., 2019).

For ease in breeding, since the 1990s, molecular markers are used to identify and for the selection of hybrid lines (Dreher et al., 2000; Gupta et al., 2010). Artificial selection can be done by a plant breeder for refining plant’s phenotype for a precise looked-for trait. Breeders also focus on crops that achieve multiple generations within a year which ultimately leads to gaining the desired phenotype faster (Van Bueren et al., 2011; Kandemir and Saygili, 2015). Molecular markers were employed to identify seven *Yr* genes for stripe rust resistance in synthetic wheat (Farrakh et al., 2016). Several markers including RFLP, AFLP, SSR, and SNP have been used to identify QTLs in rice and other crops (Oladosu et al., 2019). Single nucleotide polymorphism (SNP), a DNA marker of choice is ubiquitously present in the crop genome and is quite easy and cost-efficient (Liakos et al., 2018).

The rate of annual yield improvement for major crops ranges between 0.8 and 1.2% which must be doubled to meet the exceedingly augmented future call of plant-based goods (Li et al., 2018). With the use of new approaches, we can help boosting up the staple food crops production by improving the genetics of the crops otherwise global food security will be severely compromised in the coming two to three decades. One of the major bottlenecks in plant breeding is the time it takes to develop an improved crop variety. Molecular breeding with advanced genomic studies increases the efficiency of breeding practices and also saves time. Equated with other kingdoms, the plants are straightforwardly manipulated with a desirable trait by crossbreeding, selfing, or both because of their short-generation time and larger population size that is available for studies (Stetter et al., 2016). Recently, Lee Hickey and colleagues described the idea of “speed breeding,” which is a non-GMO approach enabling the scientists and researchers to turn over many generations in a single year and select plants with desirable traits between thousands of variations (Watson et al., 2018; Voss-Fels et al., 2019). In speed breeding technique, controlled environmental settings and extended photoperiods are achieved leading to four to six generations of crops, i.e., wheat, canola, barley, etc., in a year.

**FIGURE 2:**
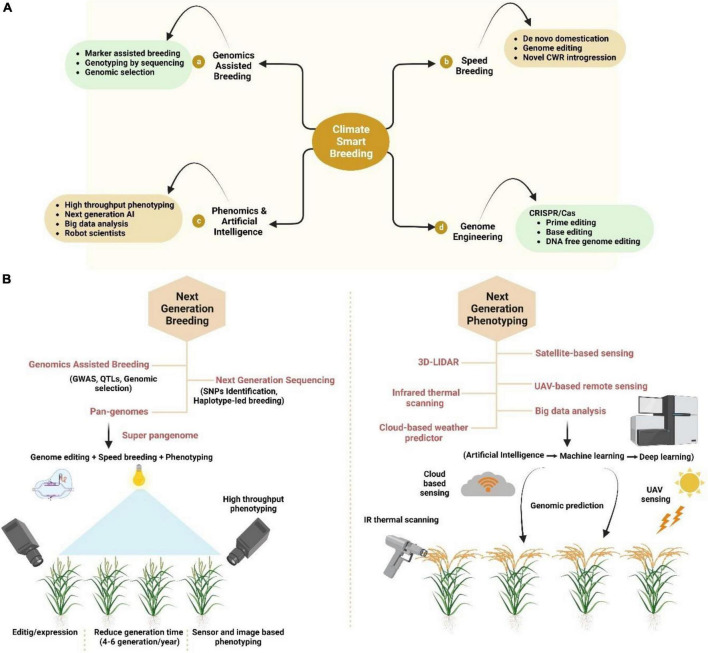
Modern breeding for crop improvement under climate change scenario. **(A)** Climate-smart breeding is the combination of (a) genomics assisted breeding, (b) speed breeding, (c) phenomics and artificial intelligence (AI), and (d) genome editing. **(B)** Next generation breeding and phenotyping tools including breeding with genomics, next-generation sequencing (NGS) and Pan genomics while phenotyping includes 3D LIDAR, satellite-based sensing, UAV-based remote sensing, cloud-based sensing, and Infrared thermal prediction. All these breeding techniques and tools help in the sustainable production of crops as well as the selecting the high yield crops.

Advances in DNA sequencing platforms and high throughput phenotyping have revolutionized the crop breeding and research opening up the genomics era of crop improvement. It has given the concept of new generation genotyping and phenotyping for crop breeding (Figures 2A,B). With the rapid advancement of next-generation sequencing (NGS) platforms, the complex genomes of many important crop species such as sorghum (730 Mbp) (Paterson et al., 2009), soybean (1115 Mbp) (Schmutz et al., 2010), barley (5100 Mbp) (Mayer et al., 2012), potato (850 Mbp) (Xu et al., 2011), and rapeseed (1200 Mbp) (Chalhoub et al., 2014) have been sequenced. Even the huge hexaploid genome of bread wheat (17000 Mbp) has been mapped with the combination of flow cytometry and synthetic mapping and next-generation sequencing technologies by enabling the chromosome-based draft genome sequence available (Mayer et al., 2014).

## Harnessing the potential of wild crop relatives for genetic diversity

Wild crop relatives (CWRs) related to the agricultural crops can enhance the adaptive capacity of the agricultural systems worldwide. They represent a pool of genetic diversity that can be used to draw new allelic variations required in breeding programs. These crop wild relatives have been extremely valuable in adapting crop varieties to changing farming practices, disease pressures, market demands, and climate conditions. The annual contribution of CWRs to the world economy is estimated to be approximately 186.3 billion USD (Tyack et al., 2020). For instance, the genes from the wild tomato increased the content of soluble solids with a worth of approximately 250 million USD.^1^ Over the past few decades, the number has significantly increased in introducing traits from the wild species into the cultivated crops mainly for overcoming the biotic and abiotic stresses. For instance, the introduction of late blight (fungal disease caused by *Phytophthora infestans*) resistance from the wild potato *Solanum demissum* and stem rust (fungal disease caused by *Puccinia graminis* ssp. *graminis*) resistance from the wild wheat *Aegilops tauschii* (Kilian et al., 2010). Generally, the primary strategy for crop improvement is a recurrent selection of wild species as a source of novel material to broaden the genetic bases of crops (Cooper et al., 2001; Moore, 2015). Wild relatives’ diversity can be classified into two major avenues; (a) “Choose first”-In this class wild species, based on phenotype and genotype for a particular trait is selected and used for crosses, while in the second class (b) “Cross first”-wide range of crosses are performed with wild species and progeny is screened for desired traits in domesticated background (Thormann et al., 2014). So, once the trait of interest has been identified in the wild genotype or individual, they need to be transferred into the crop backgrounds. Alternatively, crosses between wild and cultivated taxa are made first and their progeny, either F_1_ or later generation are screened for desired traits.

The subsequent genetic improvement led to the development of high-yielding crop varieties, many with resistance to abiotic stresses as well as pests and disease stress. The FAO estimates that approximately 75% of the genetic diversity harbored in traditional agricultural crop varieties has been lost over the past century.^2^ This important genetic loss caused by the migration of crops from their origins or modern breeding should be known as post-domestication or breeding bottlenecks (Abbo et al., 2014). There are some crucial challenges in the expansion of cultivated gene pool by using CWRs and ancestral landraces that include biological barriers to compatibility and crossability, F1 generation and backcross (BC1) sterility, reduced recombination between elite and CWR genomes, and infertility of offspring (Zamir, 2001). Careful consideration of these obstacles has opened the novel opportunities for managing the male and female sterility in the production of hybrid crops. For instance, male sterility due to the disharmony of cytoplasmic (wild) and nuclear (cultivated) genomes in interspecific crosses of vast crop species has proved to be a boon for the hybrid industry worldwide (Bohra et al., 2021).

Wild crop relatives generally express poor adaptation beyond their natural distribution range such as photoperiod sensitivity, phenological differences, and asynchronous flowering can all contribute to maladaptation to the artificial agricultural environments (Cowling, 2013; Wang et al., 2017). One of the examples here is chickpea CWRs that are collected from temperate regions show poor adaptability in tropical and subtropical regions owing to large phenological differences (Warschefsky et al., 2014). However, the perception of agronomic potential may be deceptive because an agronomically inferior CWR may contain valuable alleles for a specific trait(s). Using appropriate screening procedures, these beneficial alleles can be readily discovered in segregating population derived from wild × elite crosses (Dempewolf et al., 2017).

Genetic diversity in landraces provides a great opportunity for sustainable and improved crops. Different germplasm banks in the world play a crucial role in maintaining and sustaining the accessions of crops gathered over centuries that could be helpful in conserving the genetic resources. For instance, International Maize and Wheat Improvement Center (CIMMYT) established in 1966 runs a genetic resource program that conserves the global maize and wheat germplasm. CIMMYT holds around 150,000 wheat seeds sampled from almost 100 countries and 28,000 samples are present in the maize bank. CIMMYT launched its wheat breeding program for biofortification in 2006 and crosses among goat grass, a wild relative and wheat achieved improved disease resistance, agronomic traits, and higher zinc in wheat grains. This biofortification program is underway in different countries and hopefully will play a major role in releasing biofortified wheat that could fulfill the demand for nutrient-rich wheat in southeast Asia and other parts of the world (Singh et al., 2017). Pearl millet and its wild relative accessions have also been collected and conserved in ten gene banks in eight different countries (Sharma et al., 2021).

## Next generation sequencing and omics approaches for breeder’s arsenal

The Sequencing of more complex genomes required more effort. High content of repetitive element and ploidy level in complex genomes are the key challenges for plant sequencing projects. The availability of reference genome enables identification of large number of genes involved in biotic and abiotic stresses and also molecular markers. Re-sequencing projects are more appropriate to pre-breeding activities to identifying genomic variations and gathering information about useful polymorphisms. Various important plant species have been sequenced and their draft genomes have become available (Mosa et al., 2017). NGS based techniques have provided with the opportunity for enhanced resolution of QTLs and identification of genetic variations. High-throughput NGS in different formats have been used for crop population mapping. Some such studies have been highlighted in Table 1. With the advent of modern NGS technologies, following techniques are efficiently used for gene and QTLs discovery in crop plants.

**TABLE 1:** Bioinformatics tools utilized in modern crop breeding.

Tools and platforms used in NGS analysis for crop breeding			

Tool/Platform	Language	Characteristics	Weblinks
AutoSNP	Perl	SNP identification	https://biokeanos.com/source/autoSNPdb
Blast2GO	Linux/Windows	Genome/transcriptome annotation	http://www.blast2go.de/
CNVKit	Python/Linux	Variant Discovery in NGS data	https://cnvkit.readthedocs.io/en/stable/
DAVID	Set of annotation tools	Data annotation	https://david.ncifcrf.gov/
Galaxy	Cloud platform	NGS Data analysis	https://usegalaxy.org/
GATK	Linux	Variant Discovery in NGS data	https://gatk.broadinstitute.org/hc/en-us
InterPro Scan	Online/Linux	Domain and motif analysis	https://www.ebi.ac.uk/interpro/about/interproscan/
KEGG	Web interface/Linux	Pathway analysis	http://bioinfo.org/kobas
MapMaker	Windows	QTL analysis	https://www.softpedia.com/get/Science-CAD/MapMaker.shtml
MapQTL	Windows	QTL mapping	http://www.mapqtl.nl
MISA	Perl	SSR detection	https://biokeanos.com/source/autoSNPdb
QTLcartographer	Windows	Composite interval QTL mapping	http://statgen.ncsu.edu/
Qu-gene	Windows	Simulation for quantitative genetics	http://www.uq.edu.au/lcafs/index.html
SnpEff	Java/Linux	SNP calling	https://pcingola.github.io/SnpEff/
SNPpipeline	C++, Perl, and Python	SNP detection	http://www.icrisat.org/gt-bt/softwares_downloads.htm
SnpSift	Java/Linux	SNP filtering	https://snpeff.blob.core.windows.net/
Tassel	Windows/Linux	Association mapping	http://sourceforge.net/projects/tassel
TROLL	C++	SSR occurrence locator	http://sourceforge.net/projects/finder
**Pan-genomics**			
PanGP	Linux/Windows	Profiling analysis for the development of core genome	https://pangp.zhaopage.com/
RPAN	Linux	Rice pan-genome analysis	http://cgm.sjtu.edu.cn/3kricedb/
SplitMem	Linux	de Bruijn graph-based visualization algorithm	http://splitmem.sourceforge.net/
PanViz	Linux	Pan-genome visualization	https://github.com/thomasp85/PanViz
PGAP PGAP-X	Linux	Pan-genome cluster and evolution analysis	http://pgap.sf.net/
Pantools	Linux/Windows	Genome mapping	https://git.wur.nl/bioinformatics/pantools

**Crop databases**			

**Databases**	**Species**	**Contents**	**Weblink**

MaizeGDB	Maize	Genetic and phenotypic data	http://www.maizegdb.org/
Gramene	Arabidopsis, rye, millet, wheat, sorghum, rice, and maize	Genomic data	http://www.gramene.org/
Cottongen	Cotton	Genomic and marker information resource	https://www.cottongen.org/
Sol Genomics	Solanaceae	Network/database	https://solgenomics.net/
Soybase	Soybean	Genomic and genetic data	http://soybase.agron.iastate.edu/
TAIR	Arabidopsis	Arabidopsis genomic and transcriptomic information resource	http://www.arabidopsis.org/
plantTFDB	22 plants	Transcription factors database	http://planttfdb.gao-lab.org/

Genome-wide association studies (GWAS) lead to high resolution mapping in a larger population by offering the detection of statistically significant phenotype-genotype association based on linkage disequilibrium (LD).

Restriction site-associated DNA sequencing (RADSeq) employs NGS to scoring of several genetic markers from individuals of a population. Its more advanced and cost-effective method is GBS.

Genotyping-by-sequencing (GBS) is an efficient, cost-effective, and robust tool for implementing GWAS in crops. It also allows breeders to study genetic linage, marker detection, genomic diversity, and genomic selection in different crop breeding programs.

Bulk-segregant analysis sequencing (BSA-Seq) provides the modern combination of Bulk-segregant analysis with NGS that helps in precise identification of markers for a particular trait within a breeding population.

TILLING by Sequencing harbors both Tilling and Eco tilling approaches where NGS aids to fast discovery of induced or natural genetic variation, respectively. This technique helps in the identification of rare and novel mutations.

Mutmap exploits whole genome resequencing of different DNA pools in the segregating populations for SNP genotyping in the mutant population.

The trend to utilize robust genomics assisted breeding with the help of NGS approaches for crop breeding has been amplified over the last few decades (Table 2) and will continue with dropping costs of sequencing and increased efficiency of sequencing platforms.

**TABLE 2:** SNP and marker gene identification in crop plants by utilizing NGS (Khalid et al., 2021; Khan et al., 2021).

Crop	Targeted traits	Method	No. of SNPs/Genes
Barley	Six-rowed spring barley	GWAS	9K SNP, 3072 SNP
	Plant growth under drought	GWAS	9 K iSelect SNPs
	Hulls adherence to caryopsis	GWAS	7864 SNPs
	14 main agronomic traits	GWAS	9680 SNPs
	Seed aging and longevity	GWAS	107 SSRs
Brassica	Earliness traits	GWAS	201,817 SNPs
	Oil content	GWAS	385,692 SNPs
	Salt tolerance	GWAS	60K SNPs
	Seven yield-determining traits	GWAS	Brassica 60K
	Quantity of fatty acids	GWAS	60K SNPs
	Harvest index	GWAS	35,791 SNPs
	Seed germination and vigor	GWAS	60K SNPs
Maize	Stalk lodging resistance	GWAS	48,193 SNPs
	Seedling root architecture traits	GWAS	681,257 SNPs
	Southern leaf blight resistance	GWAS	25000 SNPs
	Kernel oil concentration fatty acid composition	GWAS	1.03 m SNPs
Cotton	Salt tolerance	GWAS	CottonSNP80K
	Fiber quality traits and yield components	GWAS	4729 SNP markers
	Oil content	GWAS	15,369 SNPs
	Drought stress	GWAS	55,060 SNPs
	Fiber quality traits	GWAS	53,848 SNPs
Rice	Agronomic traits	GWAS	32,655 SNPs
	Cooked rice texture	GWAS	147,692 SNPs
	Agronomic traits	GWAS	∼3.6 m SNPs
	Salinity tolerance	GWAS	6000 SNPs
	Low phytic acid	TILLING	ITPK
	Salt tolerance	TILLING	OsAKT1, OsHKT6, OsNSCC2, OsHAK11, and OsSOS1
	Arsenic tolerance	TILLING	ATT1
Sorghum	Forage quality-related traits	GWAS	85,885
	Plant height and architecture	GWAS	∼26,500 SNPs
Soybean	Plant height and the number of nodes	GWAS	62,423 SNPs
	Protein content	GWAS	SoySNP660k
	Photosynthetic response to low P stress	GWAS	292,035 SNPs
Sugarcane	Cane weight tillers/plant	GWAS	20 SSRs
Wheat	Wheat quality and yield-related traits	GWAS	10,172 SNPs
	Karnal bunt resistance	GWAS	13,098 SNPs
	Flour yield and alveograph quality traits	GWAS	10,802 SNPs
	Vernalization	TILLING	VRN-A1
	Karnal Hardness	TILLING	Pin a, Pin b
	Powdery mildew disease resistance	TILLING	TaMlo

## Bioinformatics databases and tools for the data analysis in crop breeding

Next-generation sequencing produces loads of data from a breeding population that can be through GWAS or GBS, etc.; therefore, after sequencing the data is analyzed using big-data handling of bioinformatics. Moreover, bioinformatics offer the tools both for forward and reverse genetics (Das, 2019).

The available user-friendly bioinformatics databases for nucleotide sequences are GenBank at NCBI, DNA Databank of Japan (DDBJ) and European Nucleotide Archive (ENA). For plants the database with genomics information is Ensemble Plants. Important tools of the data analysis for gene ontology and similarity searches are NCBI, GOA, BLAST, UniProtKB, GO, and KEGG. Data acquired from NGS sequencing platforms is handled by different bioinformatics tools which help in inferring information from the sequencing data. This information leads to establish a connection with plant phenotype and genotype for gene or marker identification (Kersey, 2019).

The tools, platforms, databases, and software often used in the data analysis in crop breeding are listed in Table 1.

## Post-genomic era in crop breeding

**FIGURE 3:**
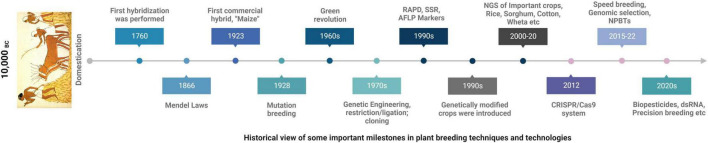
A timeline of major plant breeding discoveries for crop improvement over the years.

Since the dawn of civilization agriculture has always remained one of the topmost priorities of humans for the sustained growth and to meet the financial needs. Throughout this time different techniques have been in practice to improve the quality of food crops (Mendel, 1865; Stadler, 1928; Crabb, 1947; Welsh and McClelland, 1990; Xu, 2010; Jinek et al., 2012; Figure 3). Sustained production of crops is liable to many environmental factors; be it the biotic or abiotic stresses both negatively impact on the yield in multiple ways and is a major predicament to meet the challenge of feeding world’s increasing population which is estimated to reach at ten billion over the next three decades (Hickey et al., 2019; Varshney et al., 2021). Increasing world population combined with climate change has put scientists in a challenging position and there is a pressing need to come up with new technologies for developing climate resilient crop varieties for a sustained food production (Hickey et al., 2019). Over the last 10–15 years significant progress has been made to improve crop plants including high-throughput phenotyping system enabling us to screen large number of populations (Araus et al., 2018) and advancement of sequencing technologies made the job much easier to discover new genes for particular traits and simplified the selection criteria and to design new selection markers apart from the traditional ones (Bassi et al., 2016). One of the major quandaries in developing new crop varieties is the slow process of trait fixing and generation enhancement due to the long generation time but with the advent of “speed breeding” this problem has been alleviated by following specialized protocols and has successfully been applied on the number of crop specie including wheat barley *Hordeum vulgare*, canola (*Brassica napus*), chick pea (*Cicer arietinum*) (Ghosh et al., 2018; Watson et al., 2018).

**FIGURE 4:**
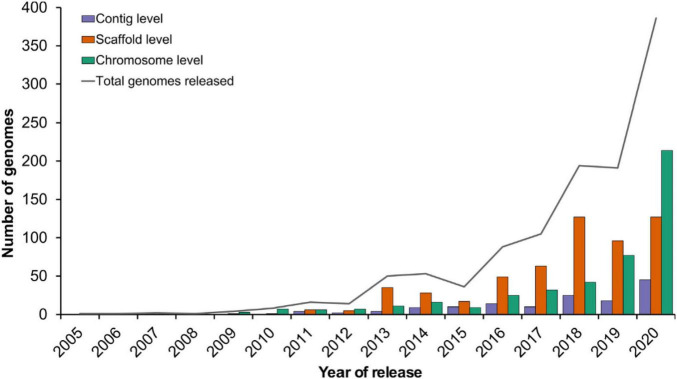
Recent trends in plant genome sequencing (Varshney et al., 2021).

Genomic assisted breeding (GAB) assisted breeders in successfully identifying allelic variation in large number of plants including the orphan and wild species followed by successful characterization and integration in the breeding programs for the crop improvement. The importance and effectiveness of genomics can be gauged from the fact that the last few years have seen a tremendous increase in its use (Varshney et al., 2021; Figure 4). This technology has been used to create countless products that not only provide protection against the biotic and abiotic factors but have also been instrumental in improving quality traits. For example, rice products including “Improved Sambha Mashuri ISM,” “Pusa Basmati” “Pusa Basmati 1121,” “Pusa Basmati 6” against bacterial blight disease (Xanthomonas oryzae pv. oryzae), “Swarna” against abiotic stress including drought and salinity (Sundaram et al., 2008; Khanna et al., 2015; Ratna Madhavi et al., 2016); Wheat products “Overlay” and “Jagger” (Kuraparthy et al., 2009); In pearl millet, “HHB 67 improved” against downy mildew disease (Rai et al., 2008); Pulse product “Pusa 10216” having drought tolerant traits. “Farnum Somerse VR 1128” were developed in United States, Canada, and Australia respectively using GAB for “grain protein content” (Mitrofanova and Khakimova, 2017).

Improving agronomic traits/quantitative traits is a difficult process because they are under control of multiple quantitative trait nucleotides (QTNs). Efficient breeding methods are required for the improvement of quantitative traits. With the advancement of genome sequencing and editing technologies the discovery and accumulation of these traits in a single genotype can now be done more easily. Due to multiple reason including climate change, insect pests resistance, a continuous surge in population, crop scientists/breeders may face serious challenges. Innovative technologies such as genome sequencing, pangenomes, genome engineering would be instrumental for the better understanding of genome structures and underlying trait architectures for precise improvement.

## Double haploid approach for climate resilience crop breeding

Haploid plants are produced naturally through parthenogenesis or elimination of the unstable genome from sperm. For the evolution of plants mixing of the genome is a key component during sexual reproduction for the success of breeding. Once the desirable genetic combination is obtained to retain this, embryos are allowed to develop from one parent followed by a doubling of chromosomes this phenomenon is called double haploid induction leads to the production of homozygous plant lines (Jacquier et al., 2020). Haploid induction in crops practically stops genetic combinations in embryos. So, when a certain genetic combination for a desirable trait is obtained it can be propagated in the next generations by producing homozygous plants. Haploid induction is done in this regard by crossing a haploid inducer line with a plant whose genetic combination is required. The genome sequence of the inducer line is not transferred to homozygous embryos. The double Haploid approach can be utilized for climate-resilient crop breeding. The induction of double haploidy in crops with favorable characters eases the passage of these characters to offspring because of the absence of dominant trait effects and new pleiotropic or gene epistasis (Prasanna et al., 2013). Various studies have been done on the incorporation of desirable genes and genetic combinations for the production of homozygous lines through the double haploid approach. In a recent study for the development of eyespot resistant lines, double haploid induction was utilized in wheat. Two wheat varieties that were highly resistant to eyespot due to the presence of the *Pch1* gene were crossed with elite wheat genotypes. Wheat varieties were crossed with maize for haploid induction and treated with colchicine solution to double the chromosome number. A total of 604 haploid plants were developed from cross combinations while 458 double haploid lines were developed after chromosome doubling. Homozygous plants were analyzed for the presence of the *Pch1* gene along with some markers associated with that gene. The eyespot resistance gene Pch1 was detected in 65 doubled haploid lines of winter wheat, in the second year of the study field trials also confirmed the incorporation of the gene (Wiśniewska et al., 2019). In another study, DH lines of winter barley developed through androgenesis showed enhanced tolerance to drought and cold stress than their parental genotypes. Also, the transcriptomic and proteomic study of these homozygous tolerant lines showed a better picture of ongoing indigenous molecular mechanisms. The genes which were not previously associated with the drought and cold stress were identified for their function. Based on this approach breeding for drought and cold resilient barley will be easy (Wójcik-Jagła et al., 2020). Double haploid lines also helped in mapping of QTLs better than heterozygous lines. While evaluation DH wheat lines against strip rust resistance seven new QTLs associated with APR genes were identified. APR genes are linked to pathogen identification and triggering immune response, identification of these associated QTLs can be helpful in determining better gene stacking (Tehseen et al., 2022). International Rice Research Institute developed rice DH line, AC-1 for the salinity tolerance that is commercialized in the Philippines and Bangladesh. Some other examples of DH rice with higher yield, commercialized in different countries are Dama (Hungry), Tanghuo 2, Tanfeng 1, Shanhua 7706 (China), Hirohikari, Kibinohana (Japan), Phalguni, Satyakrishna (India) (Samantaray et al., 2021). Also, in DH wheat lines under salinity stress QTL mapping was done and novel QTLs regarding potassium and sodium ions accumulation were detected. For the first time a novel shoot ion-independent tolerance QTL was also detected (Asif et al., 2018). In another study in Africa double haploid maize varieties were derived from commercially available hybrid maize varieties. The double haploid varieties showed remarkable improvement in yield and various other agronomically important traits. The DH hybrids also performed well under drought stress. One DH maize hybrid line showed 44.2% improved yield under drought stress and 23% improved yield under optimal water conditions as compared to best performing commercial hybrids. This superior performance of DH offspring can be marked with fixation and additive effects of favorable genes due to homozygosity of offspring (Sserumaga et al., 2018). The homozygosity in double haploid lines not only give a better picture about trait to gene function but it also enhances the action of desirable genes. Double Haploid approach along with the utilization of modern tools like speed breeding can be a key component for the development of better and sustainable agriculture.

## Exploration of pan-genomics and machine learning for understanding the crop improvement

### Pan genomics

Radical breakthroughs in high-throughput sequencing technology have unfolded new possibilities for studying genome diversity and evolution over the last two decades. Previously limited to a few reference genomes, modern technologies now allow for the analysis as well as sequencing of multiple genomes from closely related species. Undoubtedly, for many years, genomic studies were primarily based on the expensive and low-throughput Sanger sequencing, which limited large-scale population studies to a few markers and loci like simple sequence repeats (SSRs) (Zhang and Hewitt, 2003; Schmid et al., 2005). Researchers’ focus has shifted from single-genome analysis to multiple-genome analysis and population studies since the emergence of next-generation sequencing (NGS) technologies (Redon et al., 2006). As more genome sequence data become available, it becomes clear that the genomic information from a single plant species does not properly reflect the species’ diversity [4]. Since the publication of the first plant genome sequence (Kaul et al., 2000), comparative genomic studies have primarily concentrated on single nucleotide polymorphisms (SNPs) in various plant species (Zuckerkandl and Pauling, 1965; McNally et al., 2009; Lai et al., 2015). Plants have a dynamic genome as a result of various duplications for instance gene tandem duplications, rearrangement of genome, transposons activity, deletions, and recombination within populations (McClintock, 1956; Bennetzen, 2000; Yu et al., 2014; Chen et al., 2015; Gabur et al., 2019). While SNPs are frequently the focus of genomic diversity analysis, structural variation in the genome is increasingly being viewed as an essential element of genomic diversity (Zhao et al., 2018). Significant structural variations such as presence-absence variants (PAV) and copy number variants (CNV) are common in crops and play key roles in the genetic characterization of agronomical traits (Springer et al., 2009; Li et al., 2014; Lu et al., 2015).

A pan-genome is the whole set of genes found in a biological clade, as in a species. The pan-genome is further subdivided into the core genome and the variable genome (Tettelin et al., 2005). The core genome is a set of sequences or genes found in all organisms within a species, and it is the minimum genome that an individual requires for survival and basic functions (Segerman, 2012; Gordon et al., 2017; Wang et al., 2018) whereas the dispensable/variable genome is a set of dispensable genes that are either partially shared or unique to each individual (Tettelin et al., 2005). The dispensable genome, in particular, has been discovered to contain genes involved in crop growth and survival against a variety of biotic and abiotic environments such as phosphorus deficiency in rice (Gamuyao et al., 2012), head smut resistance in maize (Zuo et al., 2015), and temperature extremes (Tao et al., 2019). As a result, pangenome studies will aid in dissecting the genetics of these important agronomic traits for crop improvement (Zhao et al., 2018; Danilevicz et al., 2020).

To date, pangenomes in crop species have been generated using a variety of methodologies, such as a comparative *de novo* approach (Li et al., 2014; Schatz et al., 2014; Gordon et al., 2017; Zhao et al., 2018), an iterative assembly approach (Golicz et al., 2016b; Montenegro et al., 2017; Hurgobin et al., 2018), and the “map-to-pan” approach (Wang et al., 2018). In recent years, crop pangenomes for soybean (Li et al., 2014), maize (Hirsch et al., 2014), Brassica (Lin et al., 2014), and rice (Schatz et al., 2014) have been published. The trend toward crop pangenomes for molecular breeding instead of single sample reference genomes, will lessen sampling errors and allow for a better diversity representation (Golicz et al., 2016a). The dispensable genome of crops is found to be linked with agronomic traits, i.e., disease resistance, flowering time (Golicz et al., 2016a; Bayer et al., 2019), and environmental stress response (Hardigan et al., 2016; Hoopes et al., 2019). Graph-based pangenome was exploited to detect the missing heritability in different tomato accessions which helped it recovering 24% increase in the previously measured heritability, thus making graph-based pangenome as a suitable technique to elucidate heritability of complex traits in crop breeding (Zhou et al., 2022). CRISPR-Cas (Cong et al., 2013) technology has revolutionized plant breeding approaches by integrating them with genome editing (Scheben and Edwards, 2017, 2018; Scheben et al., 2017).

All in all, a better knowledge of the genetic diversity of the gene pool can enable trait dissection to pinpoint beneficial genetic mutations, allowing breeding programs to acquire a wide range of genetic resources to develop best breeding strategies, and eventually strengthening crop improvement to cultivate varieties with stable high yield under stressful conditions.

### Machine learning

Recent technological advancements and high throughput techniques made it possible to have ample data on plant genotypes and phenotypes which demands an extra effort to obtain meaning from these measurements and incorporate different data sets. Concurrently, machine learning has advanced rapidly and is now extensively employed in plant genotyping as well as phenotyping (van Dijk et al., 2021). More importantly, genomics does not only involve the acquisition of molecular phenotypes, but also the use of effective data mining tools to predict and describe them (Wang H. et al., 2020). Machine learning, an evolving multidisciplinary field that proposes computational and analytical elucidation for the integrative analysis of heterogeneous, large, and unstructured datasets on a Big Data scale, is becoming an important tool in biology (Ma et al., 2014; Jordan and Mitchell, 2015). Machine Learning refers to a class of computerized modeling approaches that imitate patterns from the data and make automated decisions without programming explicit rules. The chief idea behind ML is to efficiently use experiences to find core structures, similarities and dissimilarities in data to describe or categorize a new experience accurately (Singh et al., 2016). Machine learning–based algorithms are effective enough to manage enormous data sets that display high amounts of noise, dimensionality, and/or incompleteness (Liu et al., 2020; Mahood et al., 2020).

Machine learning reads the algorithms that computers use to execute tasks by learning from data rather than trailing explicit instructions (Liu et al., 2020). There are three basic approaches: supervised learning, unsupervised learning, and semi-supervised learning. The most frequently used machine learning is supervised learning, in which each example in the data set is categorized (Liu et al., 2020). Its goal is to develop a model that maps its predictors (such as DNA sequences) to target variables (such as histone marks). Some examples of supervised learning applications are: prediction of regulatory and non-regulatory regions in the maize genome (Mejía-Guerra and Buckler, 2019), predicting level of mRNA expression (Washburn et al., 2019), sequence tagging in rice (Do et al., 2018), plant stress phenotyping, prediction of polyadenylation site in Arabidopsis (Gao et al., 2018), and prediction of macronutrient deficiencies in tomato (Tran et al., 2019). Whereas, unsupervised Machine learning is based on an algorithm that does not need tags, as in case of a clustering algorithm (Libbrecht and Noble, 2015). And a semi-supervised machine-learning approach needs labels but also uses unlabeled examples (Libbrecht and Noble, 2015). Furthermore, ML based digital image have been successfully employed for assessment of diseases in crop plants. Such examples include the detection of bacterial blight disease incidence in rice (Lu et al., 2017), maize (Dechant et al., 2017), soybean (Ghosal et al., 2018), and tomato (Prabhakar et al., 2020). Similarly, digital imaging with python based ML programs was employed to assess the mosaic, spots, brown streak, mites, and nutrient deficiency in cassava (Ramcharan et al., 2019). Hyperspectral imaging technique was utilized for the detection of yellow rust in wheat (Zhang et al., 2019) and potato Y virus in potato (Polder et al., 2019).

In reality, gene-finding systems are frequently trained by utilizing a semi-supervised approach with a set of annotated genes and an unlabeled whole-genome sequence as an input. The kind of algorithm chosen by data scientists relies upon what sort of data they desire to predict. Deep learning has been employed to solve complicated biological problems in metabolomics, genomics, proteomics, transcriptomics, and systems biology, among other areas of large-scale data analysis (Xu and Jackson, 2019). One of the chief advantages of employing ML methodologies by physiologists, plant breeders, biologists, and pathologists, is the capacity to search large datasets for patterns and govern discovery by simultaneously looking at a combination of factors rather than evaluating each feature individually. ML will accelerate the development of resilient crops by identifying crucial associations that regulate biological process (Esposito et al., 2019). Deep learning is being applied in genomics at DNA, RNA, and protein level. At the DNA level, research associated with the promoter, enhancer, non-coding DNA, methylation states, TSS position, replication, *cis*-regulatory, and interaction is possible through Deep learning. At the RNA level, deep learning has been utilized to explore alternative splicing, IncRNA, microRNA, messenger RNA, and expression. Deep learning also studies DNA binding proteins, transcription factors, RNA binding proteins, and generation of protein sequence at the protein level. A Generative Adversarial Network has also been used for clarifying biological queries at various molecular levels, as discussed in Liu et al. (2020). Machine learning can be used to gain new biological insights by predicting gene function and interactions among various cellular components (Mahood et al., 2020). ML provides well-defined benefits to analyze the complicated role of gene activity in response to environmental fluctuation and in determining plant phenotypes.

Several strategies have been introduced to identify essential genes for agronomically important characters, for example, utilizing gene functions (Bargsten et al., 2014), exploiting protein interactions (Liu et al., 2017), or employing gene annotation, and sequence variation (Lin et al., 2019). Furthermore, machine learning approaches will gain more popularity for predicting crop yield, high-throughput crop stress phenotyping, and assessment of the impact of climate change (Singh et al., 2016; Crane-Droesch, 2018; Esposito et al., 2019; Tong and Nikoloski, 2021).

## New plant breeding technologies as a revolutionary toolkit for smart agriculture

In the present day, tackling total climate change, meeting human nutritional requirements, and ensuring adequate energy supplies remain resilient problems for humanity (Bevan and Waugh, 2007; Scheben et al., 2016; Hendre et al., 2019; Hunter et al., 2019). Plant breeding has always been important in human history, revolutionizing agriculture to feed the world’s continuously increasing population (Hill and Mackay, 2004). The key purpose of plant breeding is to create a genetically superior genotype that is suitable for both specific and general cultivation for increased production. In conventional breeding, farmers and crop breeders used to develop crop varieties by means of basic procedures, i.e., the presence of desired traits in plants for selection to propagate. Plant breeding evolved as an important approach for plant domestication and crop improvement around 10,000 years ago, utilizing the selection of desirable characteristics through a consistent selection process in many generations (Purugganan and Fuller, 2009).

However, crop production was inadequate to satisfy future demand, so a novel agricultural model is required, which includes cohesive systems of modern molecular breeding, various agronomic practices, and analysis of plant-microbiome interaction. As a result, climate-smart agriculture is getting prominence for developing climate-resilient crop varieties through the use of next-generation breeding strategies that can resist multidimensional stresses. And these climate-resilient crops are a crucial component concerning food and nutritional security.

Recognizing the significance of genomic resources in plant breeding programs, a massive amount of genetic data related to genes and QTLs (Quantitative Trait Loci) is obtained after the emergence of molecular biology and biotechnology (Wang and Pfeiffer, 2007). Genomics provides tools to tackle the challenges regarding food yield, quality and stability of production *via* advanced breeding techniques. Innovations in plant genomics enhance the knowledge of crop diversity at gene and species level, and an understanding of DNA markers for genetic improvement (Muthamilarasan et al., 2013, 2014).

These are some of the next-generation breeding tools that can be employed in marker-assisted selection to develop climate-resilient superior traits, combating problems of global food security.

### Genomic-assisted breeding

The use of genomics tools to improve the efficacy of plant breeding is known as GAB (Varshney et al., 2005). The GAB strategies include Marker-Assisted Backcrossing (MABC); backcrossing for beneficial alleles within elite germplasm, Marker-Assisted Recurrent Selection (MARS), and Genomic Selection (GS) that are being used in breeding programs. MABC is the most commonly used technique for improving elite varieties by introducing a few loci or Major QTLs. GAB has accelerated breeding progress across a wide range of crop species over the last 15 years, developing more than 130 publicly bred cultivars of various crops (Vogel, 2014).

Genetic mapping and QTL analysis using bi-parental or Association Mapping (AM) populations have advanced the analysis of genetic control of agricultural traits, potentially permitting MAS, QTL, and AM studies, as well as direct calculation and GS of high value genotypes for breeding programs (Kole et al., 2015). NGS combined with GWAS improves mapping resolution for accurate gene/allele/QTL location (Liu et al., 2013; Varshney et al., 2014; Kole et al., 2015). Next-generation breeding can be driven by the integration of advanced genomic technologies such as NGS and comprehensive phenotyping (Varshney et al., 2005). Genomic selection (GS) is another powerful tool for facilitating the selection of superior genotypes, speeding up the breeding cycle, and lowering the budget of breeding line development (Crossa et al., 2017). Genome-Wide Association Studies explore marker-trait build upon the large nucleotide variation found in association mapping populations.

### Genome editing

Plant breeding strategies can be revitalized by genome editing. Evidently, genome editing is creating new opportunities for accurate and faster crop modification to increase yields and guard them against diseases, pests, and abiotic stresses. The great promise of genome editing techniques is making crop breeding faster, more effective, and at a reduced cost. CRISPR genome-editing technology opens new opportunities to engineer disease resistance traits. CRISPR is expected to solve major crop improvement challenges through precise genome engineering and transgene-free applications. The introduction of next-generation CRISPR-associated (CRISPR/Cas) systems for example base editing, prime editing and *de novo* domestication, consumes the notion towards the potential of genome editing as repurposed for the improvement in crops.

Genome editing employs site-specific nucleases (SSNs) that are premeditated to bind and create a break in a particular nucleic acid site, generating double-stranded breaks (DSBs) at or close to the target site (Pickar-Oliver and Gersbach, 2019). The DNA DSBs are then repaired naturally either through homologous recombination (HR) or error-prone non-homologous end joining (NHEJ) (Miglani, 2017; Wright et al., 2018; Jun et al., 2019). These DSBs repair can be governed to obtain the ideal modifications in a sequence for instance insertions or deletions of large transgene arrays [160]. These SSNs have substantial plant breeding potential, as they present multidimensional approaches for modulating genome structure as well as function of host, such as targeted mutagenesis, gene knock-out, stacking, knock-in, and translation modulation. Browning-resistant mushrooms (Waltz, 2016b), high-amylopectin waxy corn (*Zea mays*) (Waltz, 2016a), and false flax (*Camelina sativa*) with enhanced omega-3 oil (Waltz, 2018) are recent examples of such products which were created utilizing CRISPR and authorized by the US Department of Agriculture (USDA) in quick time. As a result, CRISPR-Cas9 technology has been broadly utilized to create nutrition-improved as well as climate-resilient cereal crop cultivars (Xu et al., 2016; Shi et al., 2017; Kim et al., 2018; Razzaq et al., 2019; Raza et al., 2021). Potato is an important food crop in different countries of the world and faces challenges like drought, heat, nitrogen deficiency, bacterial diseases, insect pests, and their mediated viruses (Tiwari et al., 2022b). Solanum genus harboring genetic diversity has been explored through conventional potato breeding and can be further enriched with latest NGS based transcriptomics studies (Tiwari et al., 2020a,b,c) that would ultimately lead to the specific gene target identification for CRISPR-based genome editing (Tiwari et al., 2022a,c) providing new insights in crop improvement.

### Pan genomes

Crop pan genomics focuses on distinct genetic factors such as SNPs, mutations, and genes comprising structural variants (SVs) that govern crucial traits of interest within population. Crop pan-genome studies allow us to recover genes vanished in the reference genomes through the course of crop domestication. The accessibility of a crop’s pan-genome, which includes its CWRs, cultivated varieties and landraces, provides a well-defined scheme for collecting all information round the variations present at genotypic and phenotypic levels which allows the detection of missing, common and unique genes in the reference crop genomes (Danilevicz et al., 2020). The information of dispensable genome aids in selection of the elite crop cultivars against stresses possessing stress-responsive gene regulation (Bayer et al., 2019).

### Artificial intelligence/machine learning

Crop improvement for food security relies upon the capability to detect advantageous agricultural traits in a timely and cost-effective manner. Traditional phenotyping techniques are expensive and time consuming, so the use of high-throughput plant phenotyping (HTTP) has increased in recent years. Machine learning is basically a budding application of Artificial Intelligence, which can be characterized as cutting-edge computer-based systems which enables the machine to learn automatically and enhance its potential without being rigorously computed (Singh et al., 2016). Genomic selection allows for rapid screening of elite germplasm and accelerates crop breeding cycles (Crossa et al., 2017). Presently, genomic selection relies on advances in machine learning tools and the recovering of huge genotyping data sets associated with agronomically important phenotypic characteristics (Tong and Nikoloski, 2021).

### Speed breeding

The most intriguing technology, called as speed breeding has captured the attention of the entire world. NASA prompted a scientist from the University of Queensland to grow wheat plants in space. Speed breeding is an effective tactic for reducing crop-generation time and accelerating breeding programs for crop improvement. Speed breeding has been a revolutionary technology in agriculture and could be used to speed up crop breeding tasks for example swift gene identification, crossing, mapping populations, backcrossing, and trait pyramiding (Bhatta et al., 2021). Off-season nursery/shuttle breeding, *in vitro*/embryo culture, and double haploid technology have all been used to decrease generational interval time in various crops. Speed breeding enables speedy generation advancement by manipulating the major parameters required by the plants including, temperature, day length, and light intensity, which leads to the reduction in generation time from 2.5 to 5 as compared to the normal greenhouse and field conditions. In case of barely, chickpea, canola, and wheat 2–3 generations are usually achieved in a controlled greenhouse, however, speed breeding allows you to achieve 4–6 crop generations within a year, providing a great opportunity to develop varieties in a short duration. Other crops where speed breeding has been successfully employed are rice, soybean, sorghum, millets, rapeseed, sugarcane, tomato, and potato which is encouraging to deal with challenging food security (Hickey et al., 2019).

## Future outlook

Expanding population around the globe poses a great challenge to mankind by challenging food security issues. The major factor that is affecting the global food supply from field to product remains climate change. It throws multiple stresses on the crops including drought, salinity, pests, diseases, and other yield-related pressures. Furthermore, the inflation in food prices in recent years with the threatening economic upshot of the COVID-19 pandemic and the man-intended wars have led to disrupting the food supply chain and magnifying poverty, malnutrition, and hunger, entailing sustainable food security globally. In this scenario, Improved agriculture is the key to mitigating these concerns; therefore; biotechnology offers modern smart breeding tools for producing future smart crops. These tools have been successfully employed to run successful breeding programs; however; there is a dire need to utilize smart breeding approaches on underutilized, complex, and orphan food crops.

Crop wild relatives, already available germplasm, and landraces are potential candidates for the transfer of disease or stress-related gene pools to cultivated crops. Yield stagnancy and increased use of fertilizers in the crops might also be overcome by utilizing the CWRs, and germplasm available across the world. However, the identification of target trait genes and transferring them to cultivated crops could be very time-consuming if conventional breeding is employed alone, therefore latest gadgets of MAS, NGS, bioinformatics data processing, speed breeding, and high throughput phenomics will aid in accelerating this overall process. For instance, among cereals, Pearl millet is one of the resilient and hardiest crops that is cultivated in warm climatic regions of the world, thus has the features of climate resilience, adaptation to different ecological conditions, high nutritional value, better growth rate, and less utilization of fertilizers and irrigation. Therefore, millets can be crops of choice to transfer these beneficial attributes to other crop relatives. Moreover, the wild relatives of the millets are Pennisetum, which also has great potential to provide genetic diversity for millet and other cultivated cereals. With the advent of high throughput genomics and phenomics, now it would be possible to explore such genetic resources extensively and identify syntenic genes/QTLs in millets which can help in producing climate-smart crops to overcome food security challenges in the future.

High throughput phenomics-based smart agriculture enables the increased quantity and quality of crops with the help of artificial intelligence, machine learning algorithms, and remote sensing to explore and record the cultivation area in terms of salt, moisture levels, pH, soil quality, disease, pest or stress, and yield-related metadata. This could be very helpful in identifying the wild relatives or germplasm with target traits grown in large areas as well as for the precise selection of phenotypes among the crossed population. Genomic-assisted breeding combined with high throughput phenomics is the need of the hour for GS in less time. If a CWR or germplasm, is found harboring several good traits like disease resistance and well-adapted to hot weather, but its yield is stagnant or vice versa, then CRISPR offers the best opportunity to silence the unwanted genes in the crops.

Malnutrition is another challenge being faced by humanity, particularly in low-income countries where people are living below-standard life. Biofortified food crops could be the finest source of enriched Zn, Iron, nutrients, and vitamins. Efforts are underway to develop and commercialize biofortified wheat through breeding and it has been executed in a few countries. Nonetheless, breeding takes a long time so, as an alternative, genetically modified crops, called GMOs have been launched for crop improvement, which is not very much adored worldwide in the case of food crops because of the presence of foreign genes in plant DNA. One of the very famous illustrations is vitamin A-enriched golden rice produced in 1999 and remained under debate for two decades. It passed a rigorous process of risk assessment and got approval in the Philippines as safe rice in 2019. In this era, the new plant breeding technology tool CRISPR can aid in the deletion or insertion of the target gene for enhancing the nutritional value of staple food crops. However, the issues of complex regulatory processing, risk assessment, and public acceptance must timely be resolved, so that they could not hinder the timely crop availability to the farmers. Automated ML software and artificial intelligence programs with data analysis could potentially provide support in reducing the complicated and repeated risk assessments of the food crops.

**FIGURE 5:**
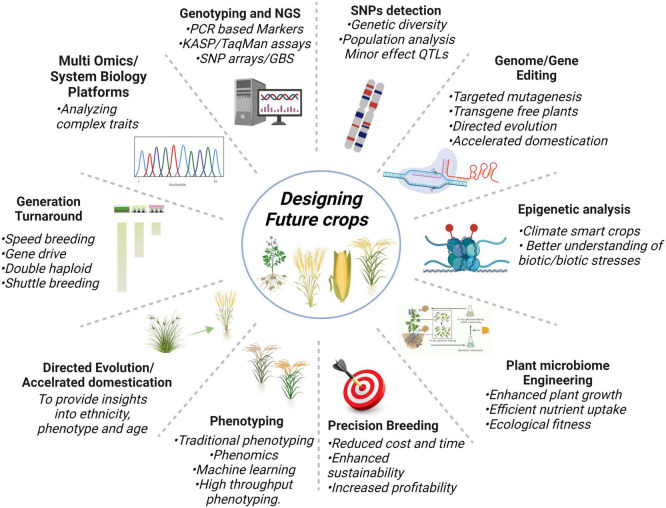
Cutting edge new plant breeding innovative technologies having the potential to turnaround the problems of food security.

Next-generation sequencing plays a crucial role to identify important genes and markers involved in a trait, for that, training of the manpower is very imperative because biological data is expanding extremely day by day. Likewise, the availability of pan-genomes of crops will aid in running improved breeding schemes, particularly in complex genome crops to elucidate the common or unique gene combinations for different traits. Nevertheless, all these technologies demand efficient computing machines and parallelization for keeping pace with growing data. The deployment of genome editing with speed breeding and phenomics can help in fast-track crop breeding. Overall, NGS-based multi-omics, genotyping techniques, genome editing, QTLome of the crop, SNP detection, plant-microbe engineering, epigenetic analysis, precision breeding, generation turnaround tools, and directed evolution are the innovative technologies as shown in Figure 5 will potentially keep helping us in designing future climate-smart food crops.

## Author contributions

MAs, SM, and RN conceived the idea. RN, HS, MM, SN, AE, and MAz wrote the first draft of this manuscript. HS and MM sketched the figures for this review. RN led the revisions and edits with the help of MF, IA, ZM, SA, MAs, and SM. All authors contributed to the article and approved the submitted version.

## Footnotes

1
www.cropwildrelatives.org/resources/in situ-conservation-manual/

2
http://www.cropwildrelatives.org/cwr/threats/
