# Supplementary material for: Smart breeding approaches in post-genomics era for developing climate-resilient food crops
Source: Front Plant Sci. 2022 Sep 16;13:972164. doi: 10.3389/fpls.2022.972164 (PMC9523482; doi:10.3389/fpls.2022.972164)
Supplement: Supplementary file 1 [file Table_1.docx]

**Supplementary Table 1.** Examples of tools aiding breeding in food crop improvement

| **Tool** | **Crop** | **Purpose** | **References** |
| --- | --- | --- | --- |
| Molecular markers | Wheat | Stripe rust resistance | Farrakh et al., 2016 |
| SSR, AFLP, RFLP and SNP | Rice | QTL Identification | Oladosu et al., 2019 |
| CWRs | Potato | Late blight resistance from the wild potato *Solanum demissum* | Kilian et al., 2010 |
| CWRs | Wheat | Stem rust resistance from the wild wheat *Aegilops tauschii* | Kilian et al., 2010 |
| CWRs | Wheat | Gaining biofortification from wild relative goat grass | Singh et al., 2017 |
| GAB | Pearl millet | Improved’ against downy mildew disease | Rai et al., 2008 |
| GAB | Rice | Bacterial blight disease resistance | Sundaram et al., 2008, Ratna Madhavi et al., 2016, Khanna et al., 2015 |
| GAB | Pulse | Drought tolerance | Mitrofanova and Khakimova, 2017 |
| Double Haploid | Wheat | Winter Wheat | Wiśniewska et al., 2019 |
| Double Haploid | Barley | Drought and cold resilience | Wójcik-Jagła et al., 2020 |
| Double Haploid | Maize | Improved yield under drought stress | Sserumaga et al., 2018 |
| NGS | Soybean | Pangenome | Li et al., 2014 |
| NGS | Maize | Pangenome | Hirsch et al., 2014 |
| NGS | Rice | Pangenome | Schatz et al., 2014 |
| NGS | Tomato | Pangenome | (Zhou et al., 2022 |
| Digital Images (ML) |  | Brown streak and mites’ detection | Ramcharan et al., 2019 |
| Digital Images (ML) | Cassava | Nutrient deficiency detection | Ramcharan et al., 2019 |
| Digital Images (ML) | Maize | Bacterial blight disease assessment | DeChant et al., 2017 |
| Digital Images (ML) | Rice | Bacterial blight disease assessment | Lu et al., 2017 |
| Hyperspectral imaging (ML) | Wheat | Detection of yellow rust | Zhang et al., 2019 |
| Hyperspectral imaging (ML) | Potato | Potato Y virus detection | Polder et al., 2019 |
| CRISPR | Maize | High-amylopectin waxy corn | Waltz, 2016a |
| CRISPR | Mushrooms | Browning-resistance | Waltz, 2016b |
| CRISPR | False flax | Enhanced omega-3 oil | Waltz, 2018 |
| Speed Breeding | Wheat, barely, chickpea, canola, soybean, sorghum, millets, rapeseed, sugarcane, tomato, and potato | Reducing generation time | Hickey et al., 2019 |
